# Bibliographical

**Published:** 1892-03

**Authors:** 


### Bibliographical.

CATCHING'S COMPENDIUM.—The second volume, that for 1891, of Catching's Compendium of Practical Dentistry, is just at hand, and in looking over it we do not hesitate to say that it is an improvement upon the first volume. The selections seem to have been made with more care, and it certainly does in the main present the leading practical matter of our journalistic literature for the year 1891 ; and it will certainly, if continued, become, in the not distant future, a ready reference book for every progressive dentist, and especially for those with whom it is an important matter to be able to promptly refer, and save the time necessary for the reading of a number of journals. These selections are made from all the journals, and no dentist, except he be engaged in editorial work, has the opportunity to read all the journals, even if he had the time and inclination ; but this compendium enables him to refer promptly to the leading practical matter that has been published. It should be found in the library of every dentist. It can be obtained of Dr. B. H. Catching, Editor and Publisher, Atlanta, Ga., or through any dental depot.

